# Editorial: Synaptopathies: from bench to bedside

**DOI:** 10.3389/fnsyn.2023.1291163

**Published:** 2023-09-27

**Authors:** Clive R. Bramham, Volkmar Lessmann, Anthony J. Hannan, Changhe Wang, Alberto Catanese, Tobias Maria Boeckers, Hongyu Zhang

**Affiliations:** ^1^Department of Biomedicine, Mohn Research Center for the Brain, University of Bergen, Bergen, Norway; ^2^Medical Faculty, Institute of Physiology, Otto-von-Guericke-University Magdeburg, Magdeburg, Germany; ^3^Florey Institute of Neuroscience and Mental Health, University of Melbourne, Melbourne, VIC, Australia; ^4^Neuroscience Research Center, Key Laboratory of Biomedical Information Engineering of Ministry of Education, School of Life Science and Technology, Institute of Mitochondrial Biology and Medicine, Xi'an Jiaotong University, Xi'an, China; ^5^Institute of Anatomy and Cell Biology, University of Ulm, Ulm, Germany; ^6^German Center for Neurodegenerative Diseases (DZNE), Ulm, Germany

**Keywords:** synapse, synaptopathies, neurodegenerative diseases, neurodevelopmental disorders, psychiatric disorders

Synapses are the fundamental structural and functional units essential for neuronal communication in the central nervous system. Dysfunction of synapses has emerged as a common characteristic of a variety of neurodegenerative, neurodevelopmental, and psychiatric diseases, as well as disorders of the spinal cord and peripheral nervous system, collectively called synaptopathies (Selkoe, 2002; Li et al., 2003; Grant, 2012; Zhang et al., 2013, 2018; Taoufik et al., 2018). Within this Research Topic, we aim at deepening our understanding of the circuit- or brain region-specific synaptic contributions to different neurological and psychiatric conditions independently from their etiological factors.

The articles in this Research Topic provide insight into GRIN2B-related neurodevelopmental disorder, synapse dysfunction during the progression of Alzheimer's disease, pathological and therapeutic mechanisms of the serotonergic system in depression and anxiety, impacts of dysfunction of the glycolytic enzyme triose-phosphate isomerase (TPI) on neuronal defects, and advances in imaging and biochemical methods for the analysis of synapses and synaptopathies. Overall, the Research Topic consists of five contributions by 25 authors: four are conceptual review articles, and one is a primary research article.

Sabo et al. provide a thorough review of GRIN2B-related neurodevelopmental disorder, with focus on current understanding of its pathophysiological mechanisms. This disorder, caused by mutations in the GRIN2B gene encoding the GluN2B subunit of the NMDA receptor, is often accompanied by intellectual disability, developmental delay, motor impairments, autism spectrum disorder, and epilepsy. The authors first describe key experimental methods for the investigation of the pathophysiology of this disease, and then discuss the impact of several distinct pathogenic GRIN2B variants on NMDA receptor properties, and on synaptic and neurodevelopmental phenotypes. These lines of evidence suggest that various pathogenic GluN2B mutants interfere with neuronal differentiation, dendrite morphogenesis, synaptogenesis, and synaptic plasticity. This review offers important insights into the pathophysiological mechanisms underlying GRIN2B-related neurodevelopmental disorder and emphasizes the importance of comparing the effects of individual pathogenic GRIN2B variants, which may lay the groundwork for personalized medicine.

Meftah and Gan give an overview of main findings, key directions and considerations in the field of synaptic alterations in Alzheimer's disease (AD). AD is a progressive neurodegenerative disease and is the synaptopathy currently receiving the most attention worldwide. Here, the malicious duo of extracellular Aβ protein (which forms plaques) and intracellular accumulation of hyperphosphorylated tau protein, along with a range of other molecular mediators, are known to be associated with the hyperexcitability of neurons early after disease onset. Subsequently, in the course of AD progression, microglia and reactive astrocyte-mediated neuroinflammation take over, continuously exacerbating synaptic functions, thereby promoting synapse loss, and eventually neuronal cell death. Thus, identifying the earliest objective signs of AD onset is instrumental to diagnose the disease before extensive synaptic and neuronal loss has occurred. Importantly, oscillatory activity (θ-oscillations, γ-oscillations and sharp-wave ripple complexes) in hippocampus and neocortex lies at the heart of learning and memory formation and could thus prove as an early biomarker for AD-related synaptic deficits. These rhythms are generated by synchronous activity of neuronal ensembles, which can be associated with the formation or retrieval of memories. This review ties together Aβ- and tau-driven synaptic dysfunction and synapse loss with altered oscillatory activity in AD mouse models and in AD patients.

Stone et al. examine the effects of glycolytic enzyme triose-phosphate isomerase (TPI) deficiency on synaptic vesicle recycling in *Drosophila melanogaster*. Many neurodegenerative diseases are associated with neuronal dysfunction caused by increased redox stress, often linked to nitric oxide (NO)-mediated post-translational changes that cause aberrant protein modifications. The authors previously identified TPI as a target for NO-mediated post-translational modifications in neurodegenerative diseases. In this article, the authors use *Drosophila* mutants expressing a missense allele of the TPI protein, M81T, resulting in an inactive mutant of TPI (*TPI*^*M*81*T*^, wstd^1^). Experimental and computational models reveal that wstd^1^ larvae display enhanced vesicle depletion rates and a significant decline in activity-dependent vesicle recycling. Moreover, TPI mutants lead to learning impairments as assessed by olfactory associative learning assays. Taken together, the data indicate functional effects of TPI impairment (wstd^1^) at the synaptic level, connecting TPI deficiency with synaptic dysfunction, a mechanism which may be involved in aggravating neurodegeneration.

Lin et al. provide latest updates on the serotonergic system in depression and anxiety. Conventional monoaminergic antidepressants share common limitations, such as slow onset and low efficacy. Different neurocircuits employ different serotonergic receptors to produce unique neurobiological effects, many of which have been the target of therapeutic drug design. In this review, the authors summarize recent findings in the cerebral localization of serotonin receptors and the pathological and therapeutic mechanisms of the serotonergic system in depression and anxiety. The authors emphasize that identifying neuronal circuit-specific signal transduction mechanisms could pave the way for producing ideal therapeutic drugs with greater efficacy and tolerability in the treatment of depression and anxiety.

Hindley et al. provide a timely and insightful review of advances in imaging synapses and synaptic proteins, methods for isolation of synaptic compartments, as well as mass-spectrometric proteomic analysis, with a focus on human synapses and synaptopathies. Using recent examples from the literature, this review first contrasts and compares methods such as immuno-electron microscopy (EM) and cryoEM with array tomography and super-resolution microscopy. Turning to molecular analysis of synaptopathies, the authors discuss the use of subcellular fractions (synaptosomes, synaptoneurosomes), fluorescence-based sorting of fractions, and their proteomics analysis using both traditional data-dependent mass spectrometric analysis, and more recently adopted data-independent acquisition, which potentially gives more proteomic coverage.

Overall, the five articles in this Research Topic reveal the relevance of synaptic dysfunction as a potential determinant of neurodevelopmental, neurodegenerative and psychiatric disorders. The identification of synapse-linked gene mutations in neurodevelopmental disorders may help develop tailored synapse-targeted therapies. In neurodegenerative diseases, such as AD and Huntington's disease, synaptic dysfunction occurs prior to overt neuronal loss, which may provide a critical therapeutic window. The advances in imaging and biochemical analysis of human synapses may help identify novel diagnostic biomarkers, which in turn aids timely treatment. It will also be exciting to see whether analysis of cortical θ- and γ-oscillations measured with routine electroencephalography (EEG) recordings might help to identify AD patients and design tailored therapeutic approaches. By linking AMPA receptors to the presynaptic release site using a synthetic synaptic organizer including cerebellin-1 and neuronal pentraxin-1, Suzuki et al. (2020) have successfully restored the number of synapses, synaptic plasticity and memory in AD models, and also reversed motor deficits in ataxia and spinal cord injury models. This highlights the potential of therapeutically targeting impaired neuronal connectivity in the treatment of neurological disorders. Developing a brain region- or neuronal circuit-specific treatment may represent a promising therapeutic strategy for the treatment of synaptopathies (Zhang and Bramham, 2020).

## Author contributions

CB: Writing—original draft, Writing—review and editing. VL: Writing—original draft, Writing—review and editing. AH: Writing—review and editing. CW: Writing—review and editing. AC: Writing—review and editing. TB: Writing—review and editing. HZ: Conceptualization, Writing—original draft, Writing—review and editing.
